# Prevalence of Birt-Hogg-Dubé Syndrome Determined Through Epidemiological Data on Spontaneous Pneumothorax and Bayes Theorem

**DOI:** 10.3389/fmed.2021.631168

**Published:** 2021-04-27

**Authors:** Marie-Eve Muller, Cécile Daccord, Patrick Taffé, Romain Lazor

**Affiliations:** ^1^Respiratory Medicine Department, Lausanne University Hospital, University of Lausanne, Lausanne, Switzerland; ^2^University Center for Primary Care and Public Health (Unisanté), DFRI/Division of Biostatistics, University of Lausanne, Lausanne, Switzerland

**Keywords:** Birt-Hogg-Dube syndrome, prevalence, pneumothorax, epidemiology, meta-analysis, Bayes theorem, gender

## Abstract

**Background:** Birt-Hogg-Dubé syndrome (BHD) is a rare inherited disorder characterized by cutaneous fibrofolliculomas, multiple pulmonary cysts, recurrent spontaneous pneumothorax (SP), and renal tumors. More than 40 years after its description, the prevalence of BHD in the general population remains unknown. This study aimed at determining the prevalence of BHD by applying the Bayes theorem of conditional probability to epidemiological data on SP.

**Methods:** We performed a meta-analysis of published data on: (1) the probability of having BHD among patients with apparent primary SP (4 studies), (2) the incidence rate of primary SP in the general population (9 studies), and (3) the probability of experiencing a SP in BHD (16 studies). Results were corrected for SP relapses, stratified by gender and year of study publication (before and after 2000), and computed with the Bayes equation.

**Results:** The probability of having BHD among patients with apparent primary SP was 0.09 (95% confidence interval: 0.07, 0.11) or 9%. It was 0.20 (0.14, 0.27) in women and 0.05 (0.04, 0.07) in men. The incidence rate of primary SP in the general population was 8.69 (6.58, 11.46) per 100,000 person-years (p-y). It was 3.44 (2.36, 4.99) per 100,000 p-y in women and 13.96 (10.72, 18.18) per 100,000 p-y in men, and was about 2 times higher in studies published after 2000 than in those published before 2000. The probability of experiencing at least one SP among patients with BHD was 0.43 (0.31, 0.54) or 43%, without gender difference. By combining these data in the Bayes equation, we found a prevalence of BHD in the general population of 1.86 (1.16, 3.00) per million, with values of 1.86 (1.02, 3.39) per million in men, and 1.88 (0.97, 3.63) per million in women.

**Conclusion:** The prevalence of BHD in the general population is about 2 cases per million, without difference between genders.

## Introduction

Birt-Hogg-Dubé syndrome (BHD) is a rare inherited autosomal dominant disorder caused by germline mutations in the tumor suppressor gene *FLCN* encoding the protein folliculin (1). Its clinical expression includes cutaneous fibrofolliculomas, multiple pulmonary cysts, recurrent spontaneous pneumothorax (SP), and renal tumors. However, BHD shows a wide phenotypic variability, and affected subjects can present with any combination of skin, pulmonary, or renal manifestations of varying degrees of severity, even within the same family. Hence, recognition of BHD remains difficult and, more than 40 years after its first description (2), its prevalence in the general population is still unknown.

One characteristic of BHD is the frequent occurrence of SP due to rupture of pulmonary cysts, which affects about half of individuals during their life, and frequently recurs. Despite the rarity of BHD, the occurrence of SP in BHD is so common that 5–10% of apparently primary spontaneous pneumothorax (PSP) in the general population appears in fact due to BHD (3–6).

To determine the prevalence of BHD, we used an indirect approach based on available epidemiological data on SP in the general population and in BHD. For this purpose, we performed meta-analyses of published studies on: (1) the probability of having BHD among patients with apparent PSP, (2) the incidence of PSP in the general population, and (3) the probability of experiencing at least one SP in BHD. Results of these meta-analyses were computed with the Bayes equation, which allows to determine the probability of an event based on prior knowledge of conditions that might be related to this event (7).

## Methods

### Literature Search

A literature search was performed in April 2020 in the PubMed electronic database. The search was limited to full-text journal articles in English, French, and German. Articles whose primary or secondary outcome met the searched items were retrieved. All articles were then reviewed to identify other studies of interest in the reference lists.

To assess the incidence of PSP in the general population, a search was performed with the Medical Subject Heading (MeSH) keyword “Pneumothorax/epidemiology.” To assess the probability of having BHD among patients with apparent PSP and the probability of experiencing a SP in BHD, a search was performed with the keywords “pneumothorax” and “Birt-Hogg-Dube” combined with the Boolean operator “AND”.

### Statistics

We expressed the Bayes theorem as follows:

$$\begin{array}{l} P\left(BHD\right)=\frac{P\left(BHD|PSP\right)\cdot P\left(PSP\right)}{P\left(PSP|BHD\right)} \end{array}$$

where *P*(*BHD*|*PSP*) is the probability of having BHD in individuals experiencing an apparent PSP in the general population, *P*(*PSP*) the prevalence of PSP in the general population, and *P*(*PSP*|*BHD*) the probability of experiencing a SP (written “PSP” in the Bayes equation) in individuals with BHD. As the prevalence *P*(*PSP*) is not directly measurable, we estimated it using the following prevalence formula (8, 9):

$$\begin{array}{l} P\left(PSP\right)≅IR\cdot \bar{D} \end{array}$$

where *IR* is the yearly incidence rate of PSP, and $\bar{D}$ is the average duration of a PSP event. This prevalence formula is valid in a steady state setting (i.e., when the total population of affected and unaffected individuals remains constant over time) and provides a good approximation when the prevalence *P*(*PSP*) is small. The value of $\bar{D}$ was based on a recently published randomized trial on the treatment of PSP, which showed that the median time of recovery for a PSP treated conservatively was 30 days, whereas it was 16 days with interventional treatment (10).

In each study, data on the number of events *E*, the number of individuals at risk at the beginning of the follow-up period, and the duration of follow-up were extracted to compute the annual incidence rate *IR* of PSP (11):

$$\begin{array}{l} IR=\frac{E}{PT} \end{array}$$

Where *PT* is the person-time product expressed in person-years (p-y). Given the small number of events in comparison to the number of individuals, *PT* was simply computed by multiplying the number of individuals by the duration of the follow-up period. The variance of *IR* was computed based on the Poisson distribution. To build a 95% confidence interval (95% CI), the log-transformation was used and the delta method was applied to compute the variance. In 4 studies (12–15), the number of individuals at risk was not reported, and the population figures were retrieved from census data available online (16–20).

As the 3 components of the Bayes equation were provided by different studies, a separate meta-analysis for each component was conducted. Except for the probability of BHD given PSP, where a fixed-effect analysis was carried out given the very small number of studies, all meta-analyses were carried out using the random-effects model. For the probability *P*(*BHD*|*PSP*) of having BHD in individuals experiencing an apparent PSP, the Freeman-Tuckey double arcsine transformation was used to ensure confidence intervals covering the appropriate [0–1] support. As the incidence rate *IR* of PSP is known to be different across genders, separate analyses were carried out for each group. Also, as all studies on PSP incidence published before year 2000 had much smaller sample sizes than those published after 2000 and had *IRs* smaller than those published after 2000, a random-effects subgroup meta-analysis was carried out within each gender, with the first group defined by studies published before 2000 and the second by those published after 2000 (21). The same approach was used for the meta-analysis of the prevalences *P*(*PSP*), as they were computed based on the *IRs*. Regarding the meta-analysis of *P*(*PSP*|*BHD*), the Freeman-Tuckey double arcsine transformation was used (22).

Finally, the pooled effect sizes estimated in each strata (defined by gender and publication date <2000 or >2000) were used to compute the prevalence of BHD for each stratum based on Bayes equation. The multivariate delta method was used to compute the variance estimate of the logit transform of *P*(*BHD*).

To compute the prevalence *P*(*PSP*), the numerator of the incidence rate *IR* should include relapses to reflect the true prevalence. For 3 of the 9 selected studies, PSP recurrences were already included in the reported numerators and the published data were directly used in the calculation. For the 6 other studies, a correction was applied to the numerators to incorporate a 29% annual recurrence rate, based on the result of a recent meta-analysis (23). The prevalence *P*(*PSP*) was computed in each stratum defined by gender, publication date <2000 or >2000, and numerator corrected to include recurrences. Results were expressed as effect size with 95% CI. To assess the robustness of results to modeling assumptions, the analyses were repeated using a fixed-effect approach instead of a random-effects (as in a random-effects approach small studies have more impact on the pooled effect-size estimate).

## Results

### Probability of BHD in Apparent PSP

The literature search identified 206 articles. Four original articles were retrieved (3–6). No additional article was found after manual review. Supplementary Figure S1 shows the flow diagram depicting the search strategy. Missing data in one study (6) were completed through correspondence with the first author. Characteristics of the studies are shown in Table 1.

**Table 1:** Studies reporting the number of patients with BHD among patients presenting with apparent primary spontaneous pneumothorax.

**References**	**Country**	**Recruited patients**	**Number of patients with apparent PSP**			**Number of patients with BHD**		
			**Women**	**Men**	**Total**	**Women**	**Men**	**Total**
Ren, 2008 (3)	China	Admitted for PSP in 2 tertiary hospitals	8	94	102	2	8	10
Johannesma, 2015 (4)	Netherlands	Admitted for PSP in one university hospital	N/A	N/A	40	1	2	3
Ebana, 2018 (5)	Japan	Admitted for PSP needing VATS in one specialized center	119	452	571	32	22	54
Toricelli, 2019 (6)	Italy	Admitted for PSP in one hospital	31*	83*	114	1	5	6

PSP, primary spontaneous pneumothorax; VATS, video-assisted thoracoscopic surgery; N/A, not available. *Retrieved from author.

**Figure 1:**
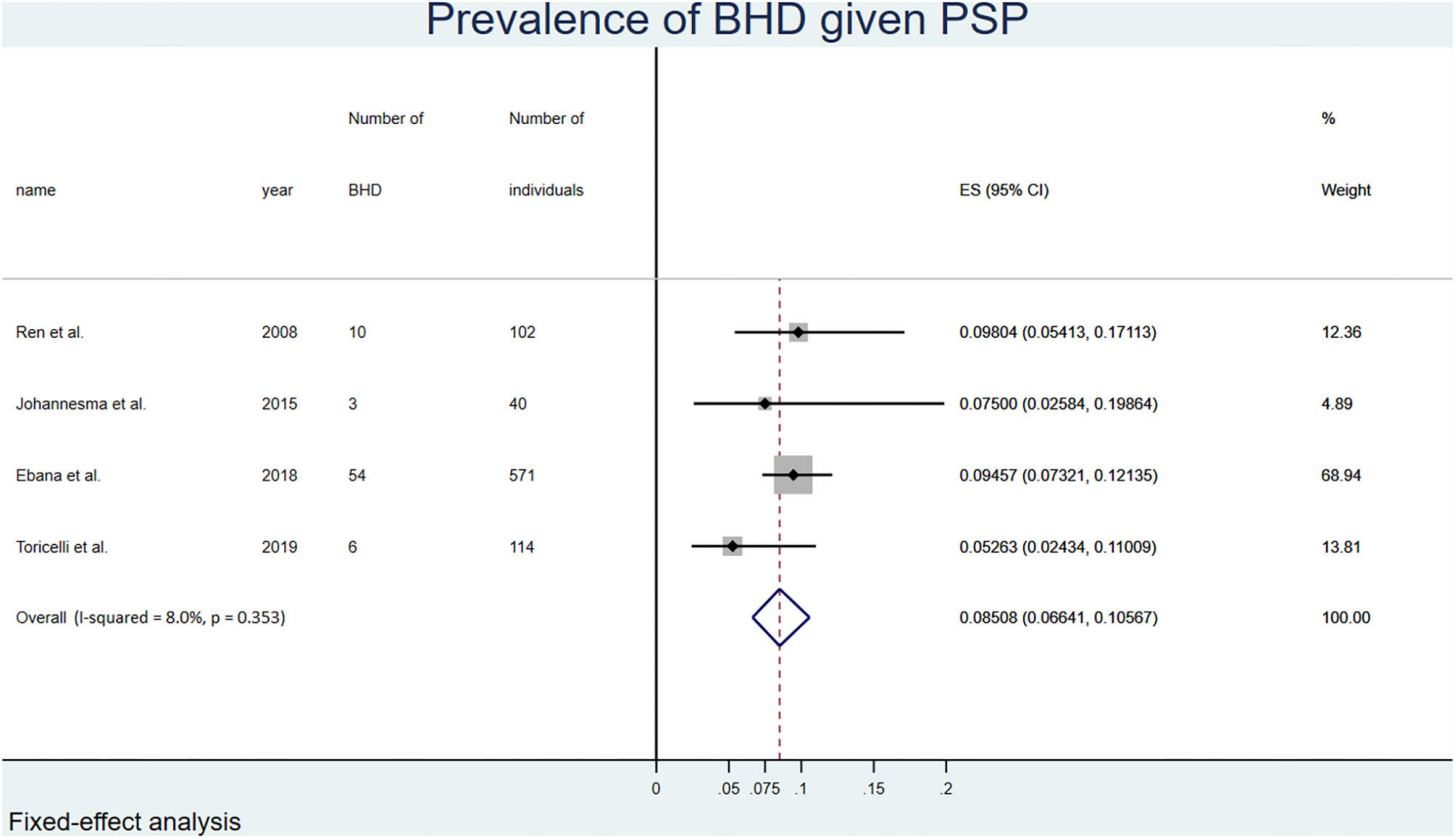
Forest plot of the overall prevalence of BHD in PSP, random-effects model.

The overall probability of having BHD among patients presenting with apparent PSP was 0.09 (0.07, 0.11) or 9%. The prevalence was 0.05 (0.04, 0.07) in men, and 0.20 (0.14, 0.27) in women (Table 2). Figure 1 summarizes the statistical results of the overall meta-analysis. The Supplementary Figure S2 shows the subgroup analyses by gender.

**Table 2:** Bayes equation's components estimated by random-effects models.

		**Women**	**Men**	**All**
Probability of BHD in apparent PSP (95% CI)		0.20 (0.14, 0.27)*	0.05 (0.04, 0.07)*	0.09 (0.07, 0.11)*
Incidence rate of PSP in the general population, per 100,000 person-years (95% CI)	overall	3.44 (2.36, 4.99)	13.96 (10.72, 18.18)	8.69 (6.58, 11.46)
	<2000	2.63 (1.31, 5.31)	10.73 (7.23, 15.92)	6.71 (4.43, 10.17)
	>2000	4.11 (2.32, 7.29)	16.95 (12.17, 23.61)	10.55 (7.40, 15.04)
Prevalence of PSP in the general population with PSP duration of 16 days, per 100,000 persons (95% CI)	overall	0.17 (0.09, 0.25)	0.65 (0.45, 0.86)	0.41 (0.27, 0.55)
	<2000	0.12 (0.00, 0.25)	0.47 (0.15, 0.79)	0.30 (0.09, 051)
	>2000	0.21 (0.09, 0.32)	0.79 (0.52, 1.07)	0.50 (0.31, 0.68)
Prevalence of PSP in the general population with PSP duration of 30 days, per 100,000 persons (95% CI)	overall	0.32 (0.16, 0.48)	1.22 (0.85, 1.60)	0.77 (0.52, 1.02)
	<2000	0.23 (0.00, 0.48)	0.88 (0.29, 1.48)	0.56 (0.17, 0.95)
	>2000	0.39 (0.18, 0.59)	1.49 (0.97, 2.01)	0.93 (0.59,1.28)
Probability of occurrence of a SP in BHD (95% CI)		0.41 (0.28, 0.54)	0.43 (0.27, 0.59)	0.43 (0.31, 0.54)
Prevalence of BHD in general population with PSP duration of 16 days, per million persons (95% CI)	overall	0.83 (0.42, 1.65)	0.82 (0.44, 1.50)	0.82 (0.50, 1.34)
	<2000	0.61 (0.23, 1.63)	0.59 (0.27, 1.27)	0.59 (0.30, 1.18)
	>2000	1.00 (0.52, 1.93)	0.99 (0.54, 1.81)	0.99 (0.62, 1.60)
Prevalence of BHD in general population with PSP duration of 30 days, per million persons (95% CI)	Overall	1.56 (0.79, 3.09)	1.53 (0.83, 2.81)	1.53 (0.94, 2.51)
	<2000	1.14 (0.42, 3.05)	1.10 (0.51, 2.38)	1.11 (0.56, 2.22)
	>2000	1.88 (0.97, 3.63)	1.86 (1.02, 3.39)	1.86 (1.16, 3.00)

95% CI, 95% confidence interval; PSP, primary spontaneous pneumothorax; SP, spontaneous pneumothorax; <2000, before year 2000; >2000, after year 2000. *Fixed-effect model.

### Incidence of PSP in the General Population

The Pubmed search retrieved 195 articles. Six articles were added after review of the reference lists. A total of 11 original articles reporting PSP incidence in the general population were retrieved. One paper was rejected because the results provided were not based on identified cases but on mathematically inferred cases. Another article was excluded because sample size and gender proportion were not given. Nine original studies were kept for meta-analysis (12–15, 24–28). Their main characteristics are shown in Table 3. Supplementary Figure S3 shows the flow diagram depicting the search strategy.

**Table 3:** Studies reporting the incidence of primary spontaneous pneumothorax in the general population.

**References**	**Country**	**Observation period**	**Recruited participants**	**Person-time**			**Number of participants with PSP**		
				**Women**	**Men**	**Total**	**Women**	**Men**	**Total**
Wynn-Williams, 1957 (24)	England	1947–1956	Admitted for PSP to the General hospital of a county town	*750,000*	*750,000*	*1,500,000*	11	59	70
Hallgrimsson, 1978 (25)	Iceland	1950–1974	Diagnosed with pneumothorax in any primary care setting or hospital in Iceland	*519,500*	*531,500*	*1,051,000*	9	33	42
Melton, 1979 (12)	USA, Minnesota	1950–1974	Diagnosed with pneumothorax in any primary care setting, hospital or at autopsy in the whole county	*923,075*	*844,150*	*1,767,225*	12	65	77
Primrose, 1984 (26)	Scotland	1976–1981	Admitted for pneumothorax to one hospital respiratory unit	*630,000*	*630,000*	*1,260,000*	11	59	70
Bobbio, 2015 (13)	France	2008–2011	Admitted for pneumothorax to any private or public hospital in France	*124,000000*	*132,000000*	*256,000,000*	12,088	38,508	50,596
Schnell, 2017 (15)	Germany	2011–2015	Admitted for PSP to any hospital in Germany AND >10 years old	*218,000,000*	*214,000,000*	*432,000,000*	12,654	40,084	52,738
Huang, 2017 (14)	Taiwan	2001–2013	Admitted for PSP to a hospital in Taiwan AND > 11 and < 40 years old	*151,000,000*	*151,000,000*	*302,000,000*	*2,836*	*16,726*	*19,562*
Hallifax, 2018 (27)	England	2015	Admitted for pneumothorax as first diagnosis to any public hospital AND > 15 years old	*23,000,000*	*22,000,000*	*45,300,000*	564	1,804	2,368
Olesen, 2019 (28)	Denmark	2009–2014	Admitted for a first episode of pneumothorax to hospital AND < 40 years old	*6,818,182*	*7,138,211*	*14,000,000*	150	878	1,028

*PSP: primary spontaneous pneumothorax. Population numbers represents the yearly population multiplied by the number of year of the observed period*.

*Values in italics: results were not available in the original paper but were re-calculated from census data*.

**Figure 2:**
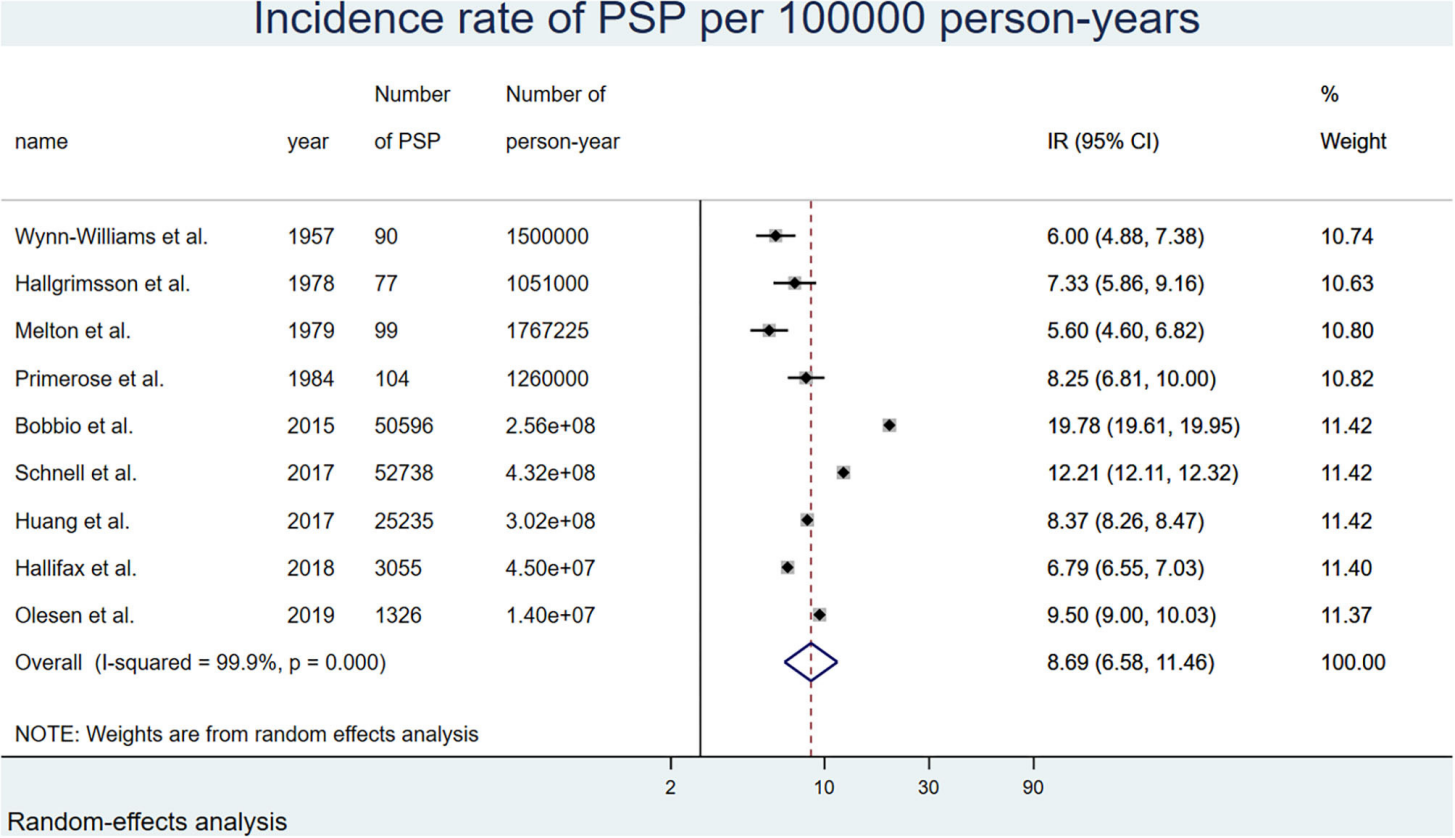
Forest plot of the overall incidence rate of pneumothorax for 100,000 person-years corrected for relapses, random-effects model.

By meta-analysis, the overall incidence rate of PSP in the general population was 8.69 (6.58, 11.46) per 100,000 p-y. Important differences appeared with gender stratification. The overall incidence rate was 3.44 (2.36, 4.99) per 100,000 p-y in women and 13.96 (10.72, 18.18) per 100,000 p-y in men. With both gender and time period stratification, women had an incidence rate of 2.63 (1.31, 5.31) per 100,000 p-y before 2000 and 4.11 (2.32, 7.29) after 2000. In men, the incidence was 10.73 (7.23, 15.92) per 100,000 p-y before 2000, and 16.95 (12.17, 23.61) per 100,000 p-y after 2000 (Table 2). Figure 2 shows the results of the overall meta-analysis. Supplementary Figure S4 show the analyses by gender and <2000/>2000 stratification.

### Prevalence of PSP in the General Population

**Figure 3:**
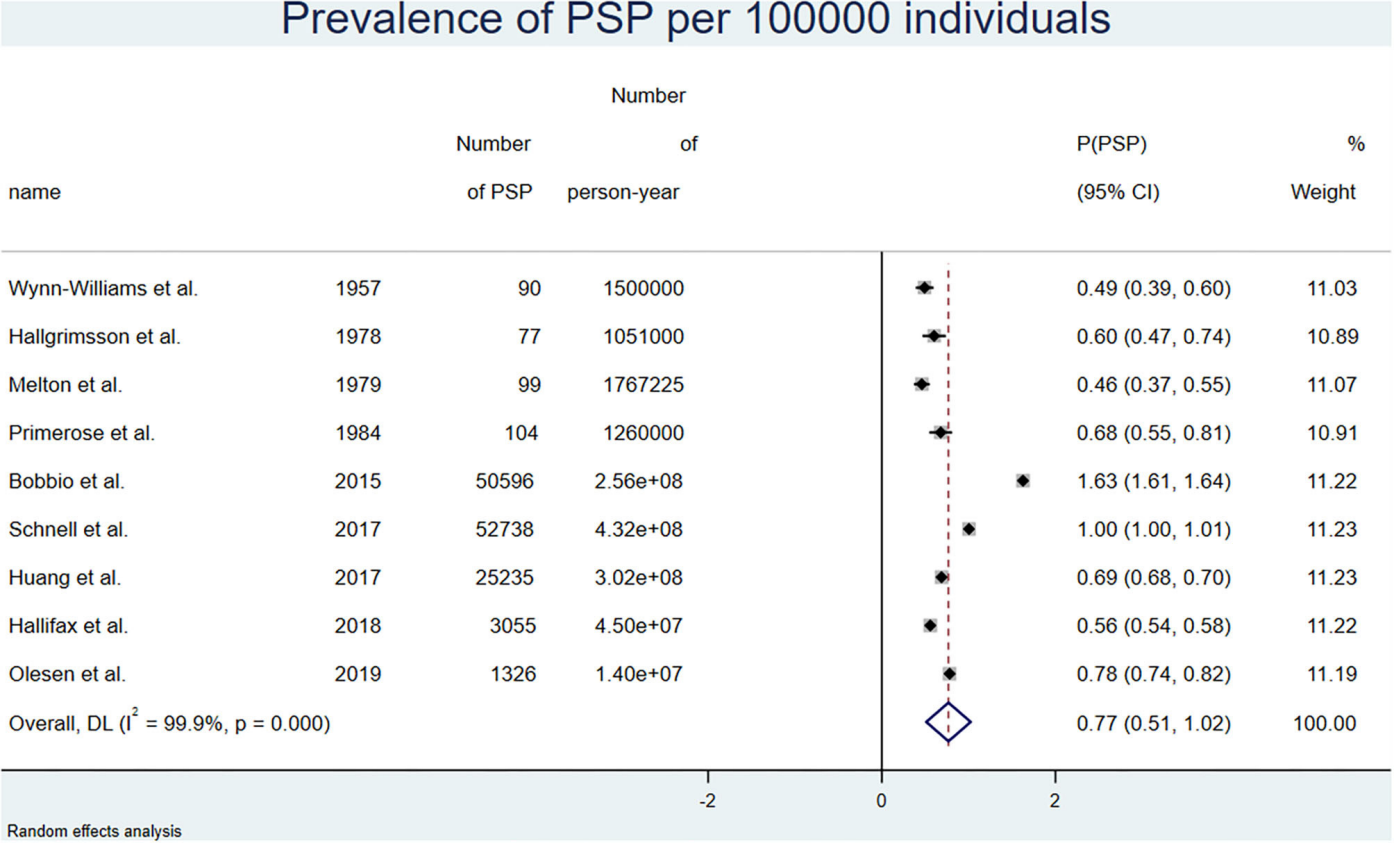
Forest plot of the overall prevalence of pneumothorax for 100,000 person-years corrected for relapses, random-effects model.

With a random-effects model, and a 30 days PSP duration, the overall prevalence of PSP in the general population was 0.77 (0.52, 1.02) per 100,000. In men, it was 0.88 (0.29, 1.48) per 100,000 before 2000, and 1.49 (0.97, 2.01) per 100,000 after 2000. In women, it was 0.23 (0.00, 0.48) per 100,000 before 2000, and 0.39 (0.18, 0.59) per 100,000 after 2000. With a PSP duration of 16 days, the prevalence was about half of these values. Results are detailed in Table 2. Figure 3 shows the results of the overall meta-analysis. Supplementary Figure S5 show the analyses by gender and <2000/>2000 stratification.

Using a fixed-effect model, the overall prevalence as well as the prevalence stratified by gender and time period were very similar to those observed with the random-effects analysis. Results are detailed in Supplementary Table S1.

### Probability of SP in BHD

The search identified 206 articles. Fifteen original articles containing data on SP in BHD were retrieved. One article was excluded because it focused only on SP in BHD after air travel (29). One original article was added after review of the reference lists (30). Another paper published by our group in June 2020 was also added (31). Thus, 16 original studies were kept for meta-analysis (3, 30–44). Their characteristics are shown in Table 4. Supplementary Figure S6 shows the flow diagram depicting the search strategy.

**Table 4:** Studies reporting the proportion of patients with BHD who experienced at least one episode of spontaneous pneumothorax.

**References**	**Country**	**Recruited patients**	**Number of patients with BHD**			**Number of BHD patients with SP**		
			**Women**	**Men**	**Total**	**Women**	**Men**	**Total**
Zbar, 2002 (32)	USA	Diagnosed with BHD	N/A	N/A	111	N/A	N/A	25
Schmidt, 2005 (33)	USA	Diagnosed with BHD and relatives	N/A	N/A	198	N/A	N/A	64
Toro, 2007 (34)	USA	Diagnosed with BHD	97	101	198	28	20	48
Leter, 2008 (36)	Netherlands	Diagnosed with BHD	15	21	36	1	3	4
Ren, 2008 (3)	China	Diagnosed with PSP and relatives	11	12	23	3	11	14
Toro, 2008 (35)	USA	Diagnosed with BHD	52	37	89	N/A	N/A	34
Kluger, 2010 (37)	France	Diagnosed with BHD	10	12	22	2	5	7
Houweling 2011 (38)	Netherlands	Suspected of BHD and relatives	N/A	N/A	115	11	17	28
Tobino, 2012 (39)	Japan	Diagnosed with BHD	14	0	14	11	0	11
Furuya, 2016 (30)	Japan	Suspected of BHD and first-degree relatives	N/A	N/A	312	N/A	N/A	230
Skolnik, 2016 (40)	Canada	One family with members diagnosed with BHD	N/A	N/A	32	N/A	N/A	13
Gupta, 2017 (41)	USA	Diagnosed with BHD	86	18	104	N/A	N/A	79
Geilswick, 2018 (42)	Denmark	BHD diagnosed cohort	57	52	109	26	17	43
Lee, 2019 (43)	R. of Korea	Suspected of BHD	8	4	12	5	3	8
Daccord, 2020 (31)	France	Diagnosed with BHD	46	50	96	30	27	57
Sattler, 2020 (44)	Germany	Diagnosed with BHD and relatives	94	103	197	38	48	86

**Figure 4:**
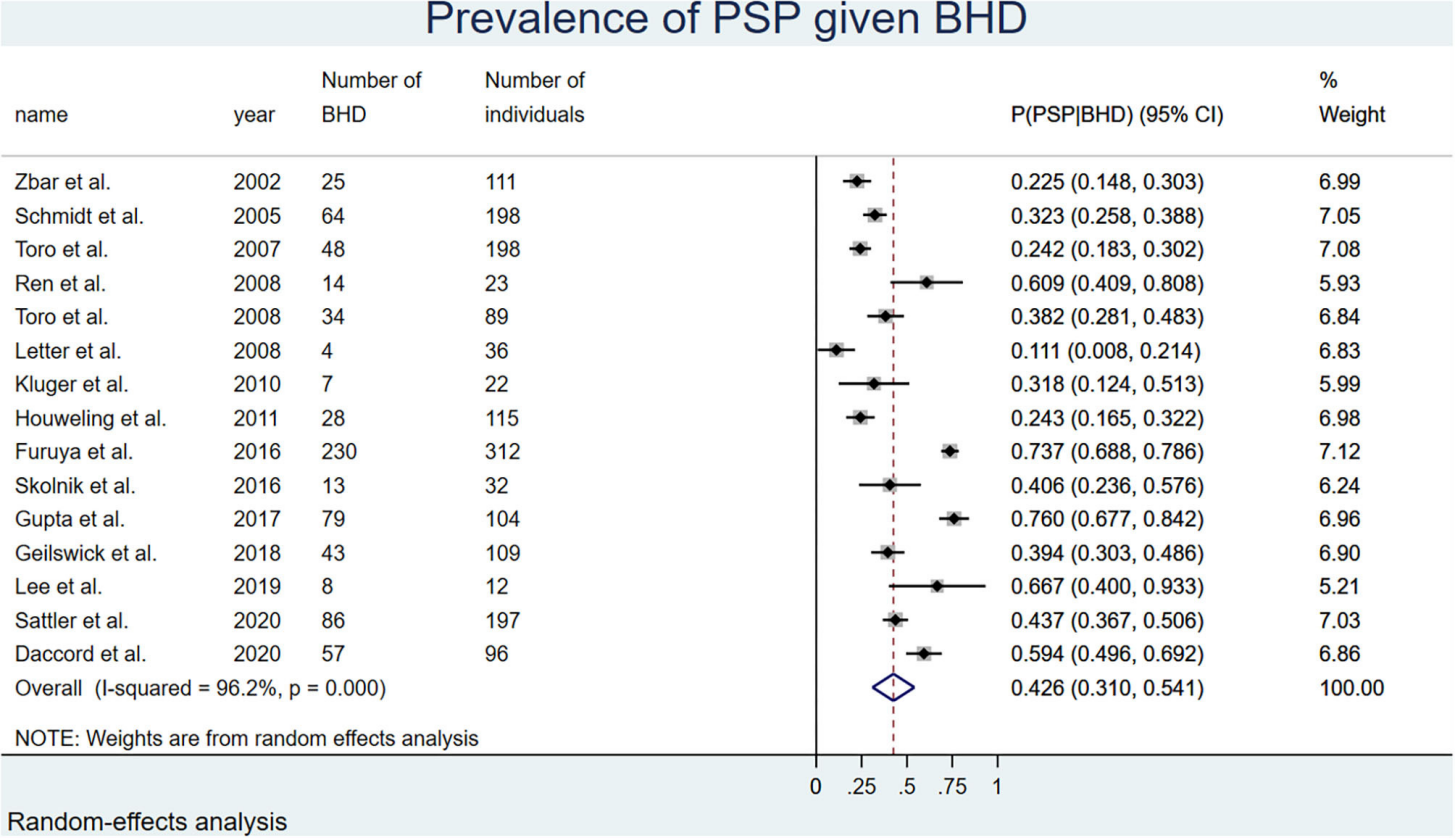
Forest plot of the overall probability of pneumothorax in BHD, random-effects model. Only 15 studies were analyzed, as one study by Tobino et al. (39) included only women.

The overall probability of ever SP among patients with BHD was 0.43 (0.31, 0.54) or 43%. There was no gender difference. Figure 4 shows the overall results of the meta-analysis. The Supplementary Figure S7 shows the subgroup analysis by gender.

### Prevalence of BHD in the General Population

To determine the prevalence of BHD in the general population, the above components were combined using the Bayes equation. Results are detailed in Table 2. We assumed that the highest accuracy would be provided by studies on the incidence of PSP published after 2000, by integrating the occurrence of relapses in the incidence of PSP, and by using a median pneumothorax duration of 30 days reflecting the natural history of the condition for the calculation of PSP prevalence. Using these assumptions, we found a prevalence of BHD in the general population of 1.86 (1.16, 3.00) per million. The prevalence by gender was 1.86 (1.02, 3.39) per million in men, and 1.88 (0.97, 3.63) per million in women. Lower figures were found when integrating studies before and after 2000, and using a median PSP duration of 16 days (Table 2).

The same calculations were made with a fixed-effect model (Supplementary Table S1). Using studies on the incidence of PSP published after 2000, taking relapses into account, and using a median PSP duration of 30 days, the prevalence of BHD was very similar to that obtained by the random-effects model with values of 1.81 (1.41, 2.31) per million for the whole population, including 2.18 (1.47, 3.23) per million in men, and 1.75 (1.20, 2.56) per million in women. Lower figures were found when integrating studies before and after 2000, and using a median PSP duration of 16 days (Supplementary Table S1).

As sensitivity analysis, we recomputed the prevalence of BHD using the Bayes formula and considering two extreme scenarios: first, a low scenario where the numerator of the formula is minimized [by using the lowest observed values of P(BHD|PSP) and P(PSP)] and the denominator maximized [by using the highest observed value of P(PSP|BHD)], then a high scenario where the numerator of the formula is maximized [by using the highest observed values of P(BHD|PSP) and P(PSP)] and the denominator minimized [by using the lowest observed value of P(PSP|BHD)]. For the low scenario, we found *P*(BHD) = 0.3 per million individuals, and for the high scenario *P*(BHD) = 14.4 per million individuals.

In summary, the prevalence of BHD in the general population was about 2 cases per million, and was equally distributed among men and women.

## Discussion

In this study, we took advantage of available data on epidemiology of pneumothorax to determine the prevalence of BHD in the general population, using an indirect approach based on Bayes equation. We performed meta-analyses of published studies to assess each component of the Bayes equation. We found a prevalence of BHD in the general population of about 2 cases per million, without differences between men and women. To our knowledge, this is the first study to determine the prevalence of BHD.

### Probability of Having BHD in Individuals With Apparent PSP

The first component of the calculation used in this study was the probability of having BHD in individuals presenting with apparent PSP. Only 4 studies addressing this issue were available, with number of PSP ranging from 40 to 571, and number of BHD among these cases ranging from 3 to 54 (3–6). The proportion of BHD among patients with apparent PSP ranged from 5 to 10% in individual studies, with a pooled value of 9%. Although a recruitment bias with enrichment in BHD patients may have occurred in studies performed in tertiary hospitals and a Pneumothorax Research Center (5), this suggests that BHD is not rare among patients presenting with apparent PSP, and should be carefully looked for in this population. One simple measure to screen for BHD is to systematically inquire about a family history of pneumothorax in patients presenting with apparent PSP. Familial pneumothorax accounts for about 10% of apparent PSP (45), with BHD being the most common cause (46). Indeed, 2 studies showed a prevalence of BHD of 64–86% in patients presenting with apparent PSP and a positive family history of pneumothorax (5, 6). It is therefore recommended to systematically look for lung abnormalities and a genetic cause, especially BHD, in any individual presenting with apparent PSP, and even more in case of a positive family history of pneumothorax (47, 48).

In the 3 studies with available gender data, we found a meaningful difference between genders in the probability of having BHD among patients presenting with apparent PSP, with a rate of 5% in men and 20% in women. Although this finding has to be interpreted with caution due to limited number of studies and small sample sizes, it suggests that the distribution of causes of apparent PSP differs between men and women. Indeed, the incidence of PSP in the general population is known to be higher in men (49), with smoking being the main other contributing factor. Consequently, true PSP appears more frequent in men, and SP due to BHD accounts for a lower proportion of apparent PSP in this population. In contrast, true PSP is less common in women, and apparent PSP in this population may be due to a greater extent to BHD, and to other diseases specific to women such as lymphangioleiomyomatosis (LAM), and catamenial pneumothorax associated with endometriosis. Indeed, LAM has been estimated to account for 5–30% of apparent PSP in women (50), whereas catamenial pneumothorax is estimated to be its cause in 25–31% (51, 52). Thus, the likelihood of finding an underlying cause is higher in women with apparent PSP as compared to men, and should prompt to carefully look for such a cause in the female population.

### Incidence and Prevalence of PSP in the General Population

For the second component of the Bayes equation, the incidence and the prevalence of PSP in the general population had to be determined. Regarding incidence, the 9 studies addressing this issue were performed in 3 different continents (North America, Europe, Asia), and the observation periods covered a large time span between 1947 and 2015. Important differences in PSP incidence were observed between studies performed before 2000 and those performed after 2000, the latter consistently showing a higher incidence both in women and men. As a true increase in incidence over time appears unlikely, we believe that the observed differences are due to more comprehensive case finding and larger sample size in more recent studies. Indeed, the 4 oldest studies, published between 1957 and 1984, were performed at a regional level (county, island, or a region smaller than a country) (12, 24–26). In contrast, the 5 most recent studies, published between 2015 and 2019, were performed at a national level and included much larger samples (13–15, 27, 28). Also, they were based on national registries of hospitalizations or medical care networks, which allowed to retrieve data more precisely and at a larger scale than the smaller studies performed decades ago. We thus considered that the true incidence of PSP was better appraised in recent studies, and chose to use the data of this subgroup for subsequent calculations.

Three studies included relapses (13, 15, 26), resulting in a higher overall incidence as compared to studies not including this parameter (12, 14, 24, 25, 27, 28). After applying a 29% correction factor to the latter studies, the difference was blunted. We therefore chose to take relapses into account to appraise the true incidence of PSP with the best possible accuracy. This value of 29% was based on a recent meta-analysis on the incidence of PSP (23).

PSP is an acute disease resulting in most cases in complete resolution. Consequently, the prevalence of PSP is usually not a relevant issue clinically, and no data on this parameter were found in the literature. We therefore used data provided by a recent interventional study comparing the outcomes of PSP treated with chest tube vs. observation (10). In this study, the median time to spontaneous resolution of PSP was 30 days, whereas it was 16 days with interventional treatment. Both results were used to calculate the prevalence of PSP. However, we considered that a duration of 30 days reflecting the natural history of the disease was more appropriate to determine a natural phenomenon such as the prevalence of BHD.

### Probability of SP in Individuals With BHD

The third component used in the Bayes equation was the probability of having SP in patients with BHD. The meta-analysis included 16 studies with available data on SP. We found a probability of having at least one SP in BHD of 43% (95%CI 0.31–0.54). The reported prevalence rates of SP tended to be higher in pulmonary cohorts (range: 42–76%) (29, 30, 53) than in renal/dermatologic cohorts (range: 23–38%) (32–35, 38), probably due to selection bias. This meta-analysis allowed to appraise more accurately the true probability of SP in BHD independently of its main clinical presentation, and to overcome the selection bias of individual studies. Our analysis confirmed that the occurrence of SP in BHD has no gender predilection, with similar SP rates between men and women of 43 and 41%, respectively.

### Prevalence of BHD

The BHD prevalence determined in this study varied moderately according to assumptions made regarding period of publication of studies on PSP incidence, and duration of PSP used to calculate PSP prevalence. The value of about 2 cases per million was the highest among the possible outcomes, but we believe that it is the most reliable. A value of about one case per million was found by pooling all available studies on PSP incidence (both before and after year 2000) and using the lowest value of PSP duration of 16 days. However, all values remained within the same degree of magnitude, thus reinforcing the validity of our observations. We primarily chose to use the random-effects model, but very similar results were observed with the fixed-effect model, demonstrating that our results were independent of the method used. We did not find any previous publication on BHD prevalence for comparison. Very close to our findings, an estimated prevalence of BHD of 1–9 cases per million is mentioned in the Orphanet database, but how this value was determined is not specified (54). Our findings confirm that BHD is a rare disease, with a prevalence similar to or even lower than LAM, which has been estimated to occur in 2.6–7.8 per million women (55–57).

Our study has limitations. The number of studies reporting the probability of BHD in apparent PSP was small, and the number of patients included was also limited. Data about the average age of participants was incomplete in several studies and it was not possible to stratify further the analyses by age. Goodness-of-fit was difficult to assess given the still important heterogeneity even after stratifying and the uselessness of the funnel plot in this context with proportions as outcomes (58). Nevertheless, we believe that stratifying by gender and publication date already accounted for a part of the heterogeneity and provided reasonable effect sizes.

Two components of the Bayes formula exhibited high residual heterogeneity, the prevalence of PSP in the general population and the probability of SP in BHD patients. Unfortunately, most of the heterogeneity could not be explained by the two variables used (before/after year 2000 and gender) to perform the stratification, and no other study variables (such as age) were available to further stratify.

There are several possible explanations for the high residual heterogeneity of the prevalence of PSP in the general population. First, the selected studies have been carried-out in geographic subpopulations that differed importantly regarding the incidence rate of PSP in the general population (as exemplified by the study of Bobbio et al. reporting an IR of 19.78 per 100,000 p-y and that of Melton et al. with an IR of 5.6). This heterogeneity might have resulted from a different exposure to risk factors of PSP such as smoking, air pollution, meteorological conditions, genetic background, or socio-demographic characteristics. Second, different definitions of primary and secondary SP have been used in the various studies, as shown in Table 3. Third, criteria used to select the individuals and recruitment settings might also have differed across studies. A fourth source of heterogeneity of the prevalence of PSP, which we have not investigated, is the possible variability in the duration of a PSP episode across studies.

Regarding heterogeneity of the prevalence of SP in BHD patients, it could be due to different settings of patient enrollment based on the presenting clinical picture (pulmonary vs. cutaneous or renal involvement). Additionally, the risk of SP in BHD may depend on genetic factors having a variable distribution in different subpopulations. For example, genetic variants associated with multiple pneumothorax have been recently identified in European BHD patients (44).

Altogether, our results show that the incidence of PSP in the general population and the probability of SP in BHD are not uniform, and it is likely that there are subpopulations more exposed to the risk of developing these conditions. The end impact of this on the prevalence of BHD in the general population is difficult to apprehend. There might also be subpopulations more exposed to the risk of developing a BHD syndrome due to genetic backgrounds, but it is difficult to answer this question based on our meta-analyses, as a variation in the numerator of the Bayes formula may be compensated by another in the denominator.

In summary, in this first approach of BHD epidemiology, we found a prevalence of BHD of about 2 cases per million, confirming the rarity of this disorder, and the equal distribution between men and women suggested by observational case series. We also believe that the method used in this study provides a new approach to determine the epidemiology of other rare diseases.

## Data Availability Statement

The raw data supporting the conclusions of this article will be made available by the authors, without undue reservation.

## Author Contributions

RL and PT: study design. MEM: data collection. PT: statistical analyses. MEM, RL, and PT: data interpretation. MEM, CD, RL, and PT: manuscript writing, revision and final approval of the last version. All authors contributed to the article and approved the submitted version.

## Conflict of Interest

The authors declare that the research was conducted in the absence of any commercial or financial relationships that could be construed as a potential conflict of interest.

## Supplementary Material

Supplementary Figure S1 Flow diagram depicting the search strategy regarding probability of BHD in apparent PSP.

Supplementary Figure S2 Forest plot of the prevalence of BHD in apparent PSP, random-effects model, stratified by gender (**A**, in males and **B**, in females).

Supplementary Figure S3 Flow diagram depicting the search strategy regarding incidence of PSP in the general population.

Supplementary Figure S4 Forest plot of the incidence rate of PSP for 100,000 person-years with correction for relapses, random-effects model, stratified by gender and period <2000/>2000 (**A**, in males and **B**, in females). Note: due to software features, the values shown in this figure are slightly different from those of the text, as the between-study variance was calculated by the DerSimonian & Laird method, whereas in the text it was computed by restricted maximum likelihood.

Supplementary Figure S5 Forest plot of the prevalence of PSP for 100,000 person-years with correction for relapses, random-effects model, stratified by gender and period <2000/>2000 (**A**, in males and **B**, in females). Note: due to software features, the values shown in this figure are slightly different from those of the text, as the between-study variance was calculated by the DerSimonian & Laird method, whereas in the text it was computed by restricted maximum likelihood.

Supplementary Figure S6 Flow diagram depicting the search strategy regarding probability of SP in BHD.

Supplementary Figure S7 Forest plot of the prevalence of SP in BHD, random-effects model, stratified by gender (**A**, in males and **B**, in females). Due to software features, the values shown in this figure are slightly different from those of the text, as the between-study variance was calculated by the DerSimonian & Laird method, whereas in the text it was computed by restricted maximum likelihood.

Supplementary Table S1 Bayes equation's components estimated by fixed-effects models.

## Abbreviations

BHD: Birt-Hogg-Dubé syndrome
PT: person-time
SP: spontaneous pneumothorax
PSP: primary spontaneous pneumothorax
P: prevalence
IR: incidence rate.
