# Supplementary material for: Prevalence of Birt-Hogg-Dubé Syndrome Determined Through Epidemiological Data on Spontaneous Pneumothorax and Bayes Theorem
Source: Front Med (Lausanne). 2021 Apr 27;8:631168. doi: 10.3389/fmed.2021.631168 (PMC8111214; doi:10.3389/fmed.2021.631168)
Supplement: Supplementary Figure S1 — Flow diagram depicting the search strategy regarding probability of BHD in apparent PSP. [file Data_Sheet_1.pdf]

Prevalence of Birt-Hogg-Dubé syndrome determined through epidemiological data on spontaneous pneumothorax and Bayes theorem

SUPPLEMENTARY MATERIAL

Marie-Eve MULLER[1]
Cécile DACCORD[1]
Patrick TAFFÉ [2]
Romain LAZOR[1]

[1]Respiratory Medicine Department, Lausanne University Hospital, University of Lausanne, Lausanne, Switzerland.
[2]University Center for Primary Care and Public Health (Unisanté), DFRI/Division of Biostatistics, University of Lausanne, Lausanne, Switzerland

Table of contents:

Supplementary Figure S1 page 2
Supplementary Figure S2 page 3
Supplementary Figure S3 page 4
Supplementary Figure S4 page 5
Supplementary Figure S5 page 6
Supplementary Figure S6 page 7
Supplementary Figure S7 page 8
Supplementary Table S1 page 9

Supplementary Figure S1

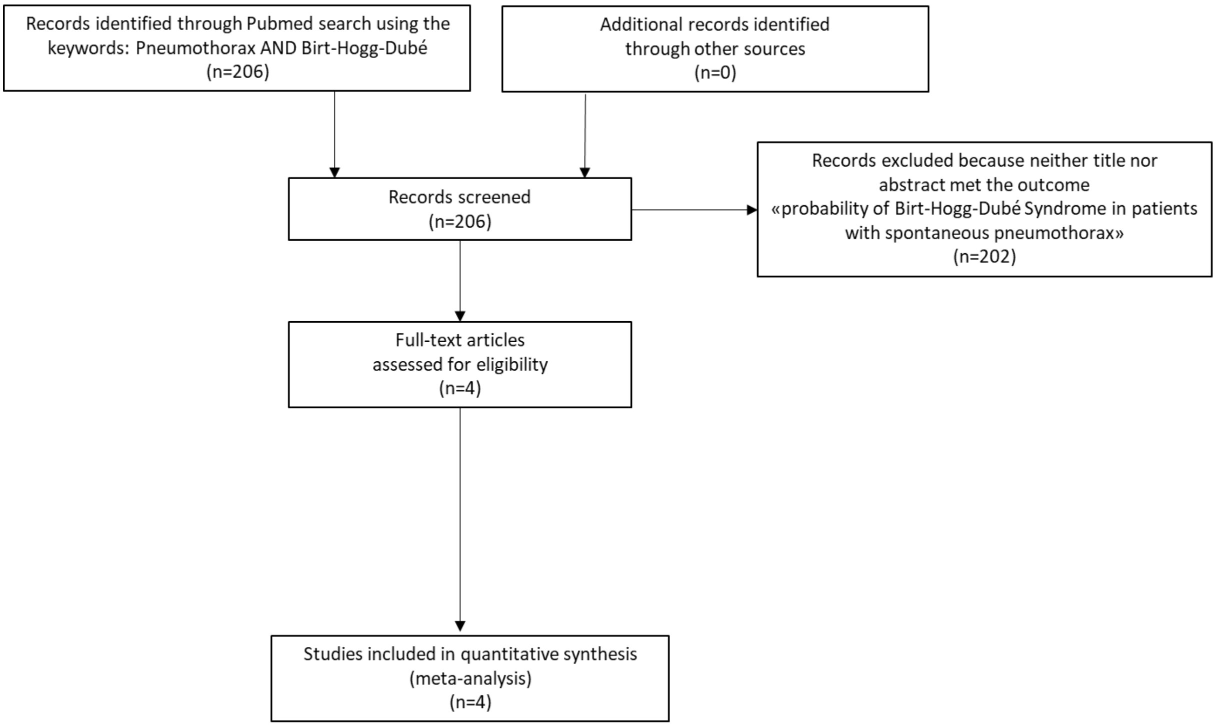

Flow diagram depicting the search strategy regarding probability of BHD in apparent PSP.

Supplementary Figure S2

A)

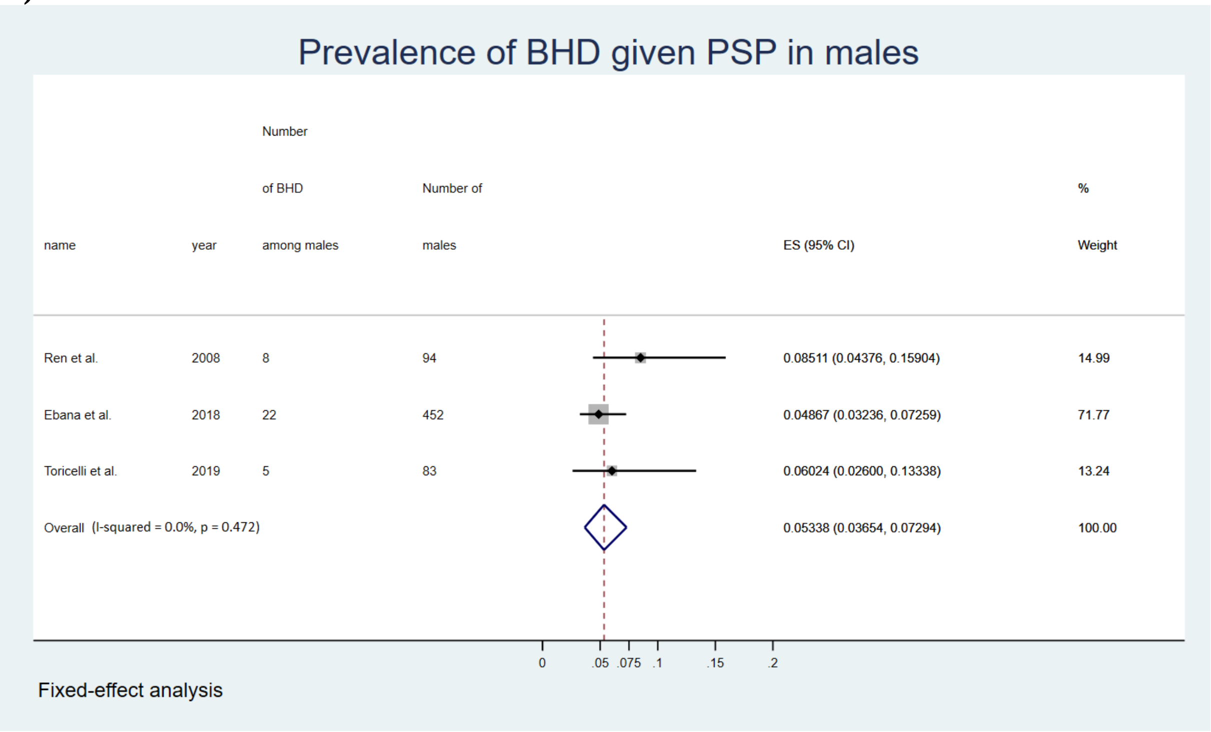

B)

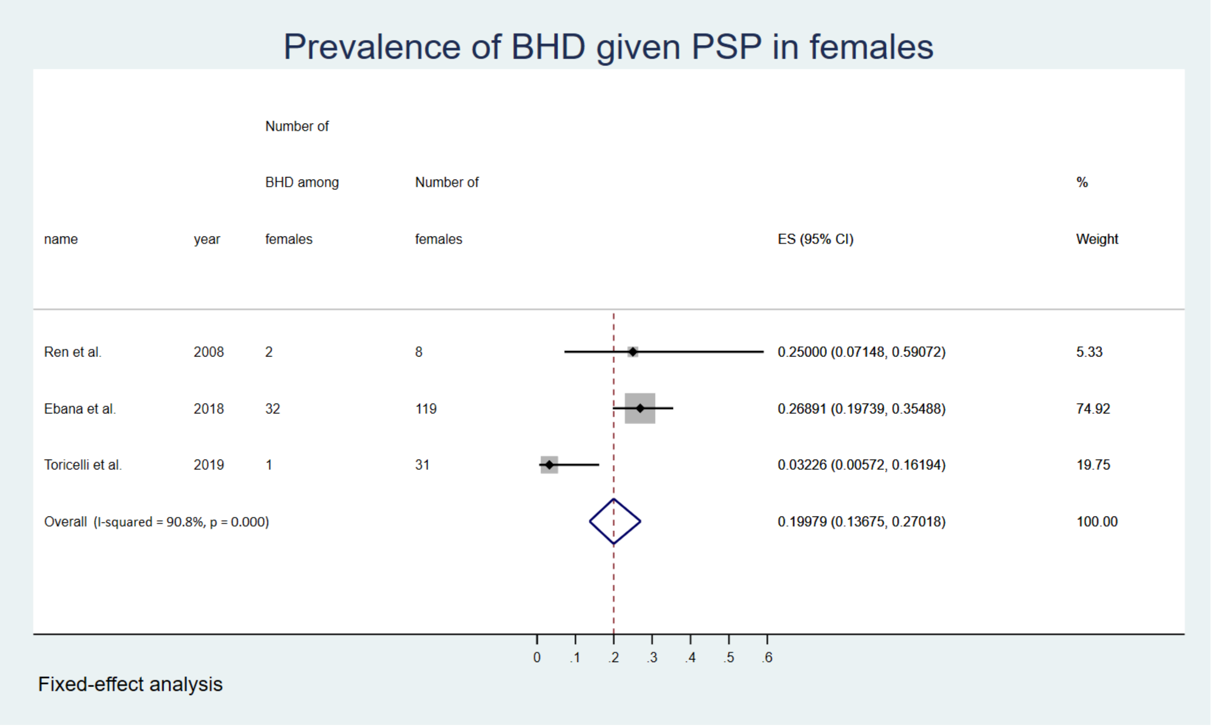

Forest plot of the prevalence of BHD in apparent PSP, random-effects model, stratified by gender (A, in males and B, in females).

Supplementary Figure S3

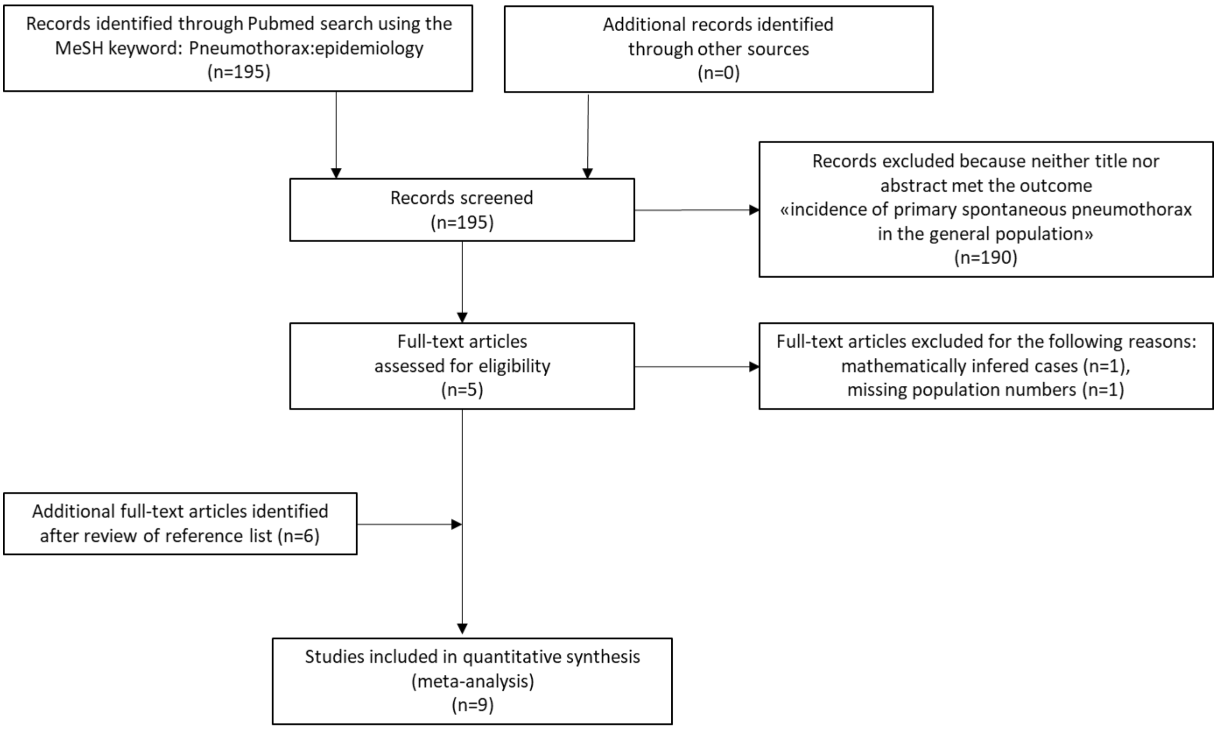

Flow diagram depicting the search strategy regarding incidence of PSP in the general population.

Supplementary Figure S4
A)

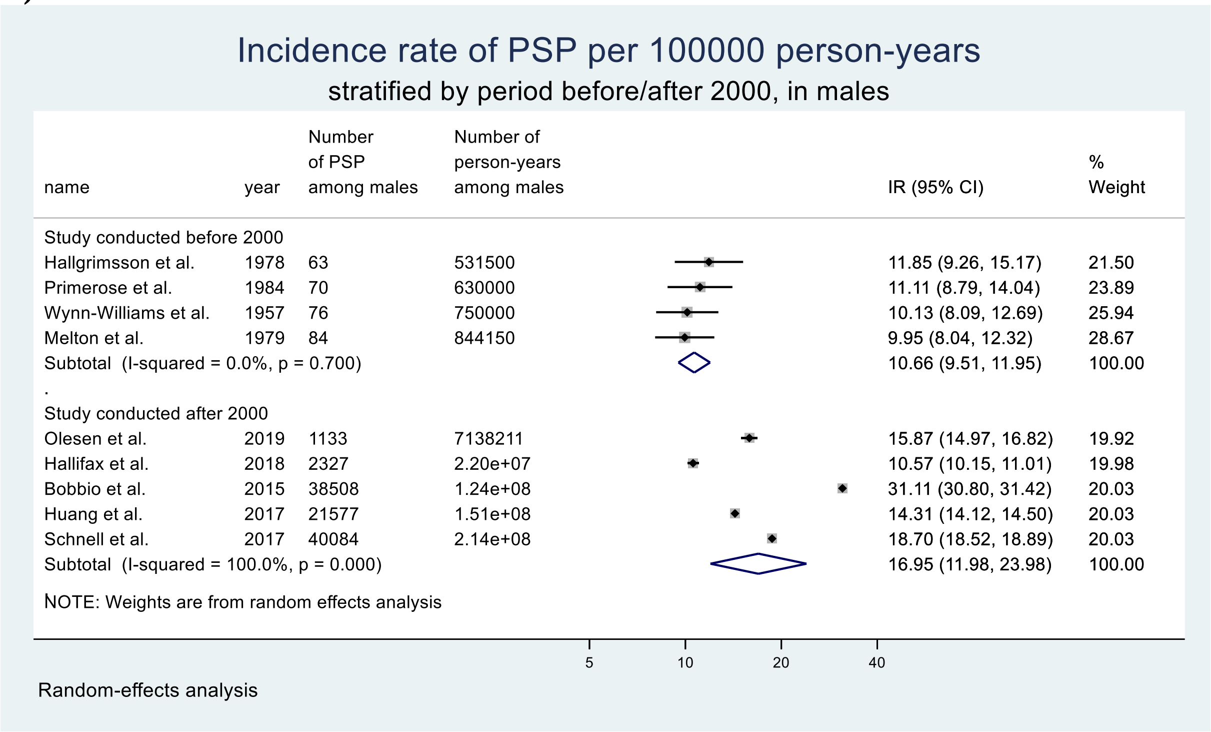

B)

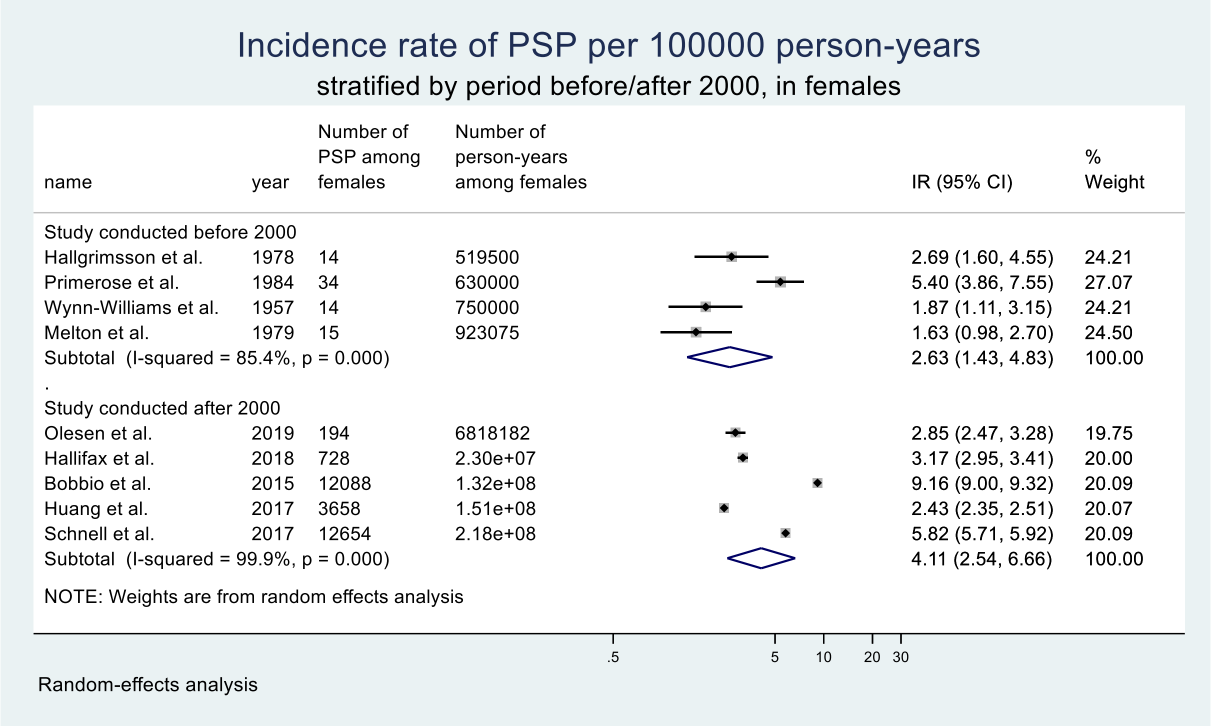

Forest plot of the incidence rate of PSP for 100,000 person-years with correction for relapses, random-effects model, stratified by gender and period <2000/>2000 (A, in males and B, in females). Note: due to software features, the values shown in this figure are slightly different from those of the text, as the between-study variance was calculated by the DerSimonian & Laird method, whereas in the text it was computed by restricted maximum likelihood.

Supplementary Figure S5
A)

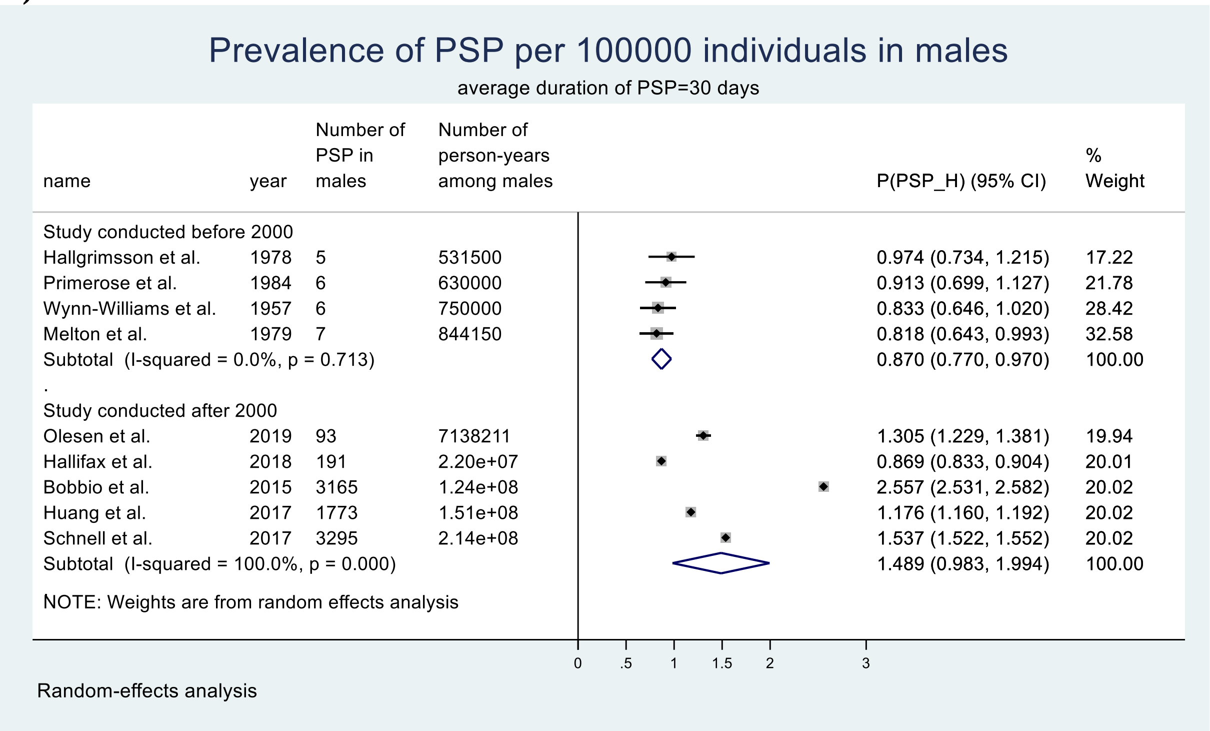

B)

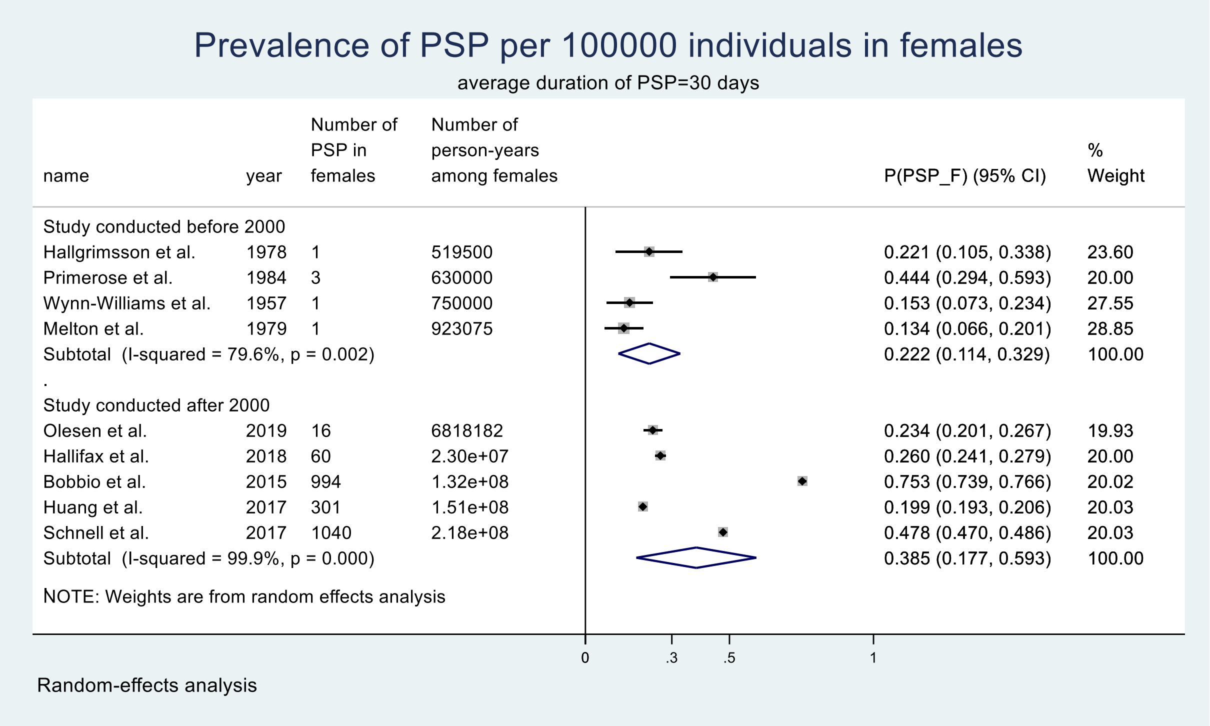

Forest plot of the prevalence of PSP for 100,000 person-years with correction for relapses, random-effects model, stratified by gender and period <2000/>2000 (A, in males and B, in females). Note: due to software features, the values shown in this figure are slightly different from those of the text, as the between-study variance was calculated by the DerSimonian & Laird method, whereas in the text it was computed by restricted maximum likelihood.

Supplementary Figure S6

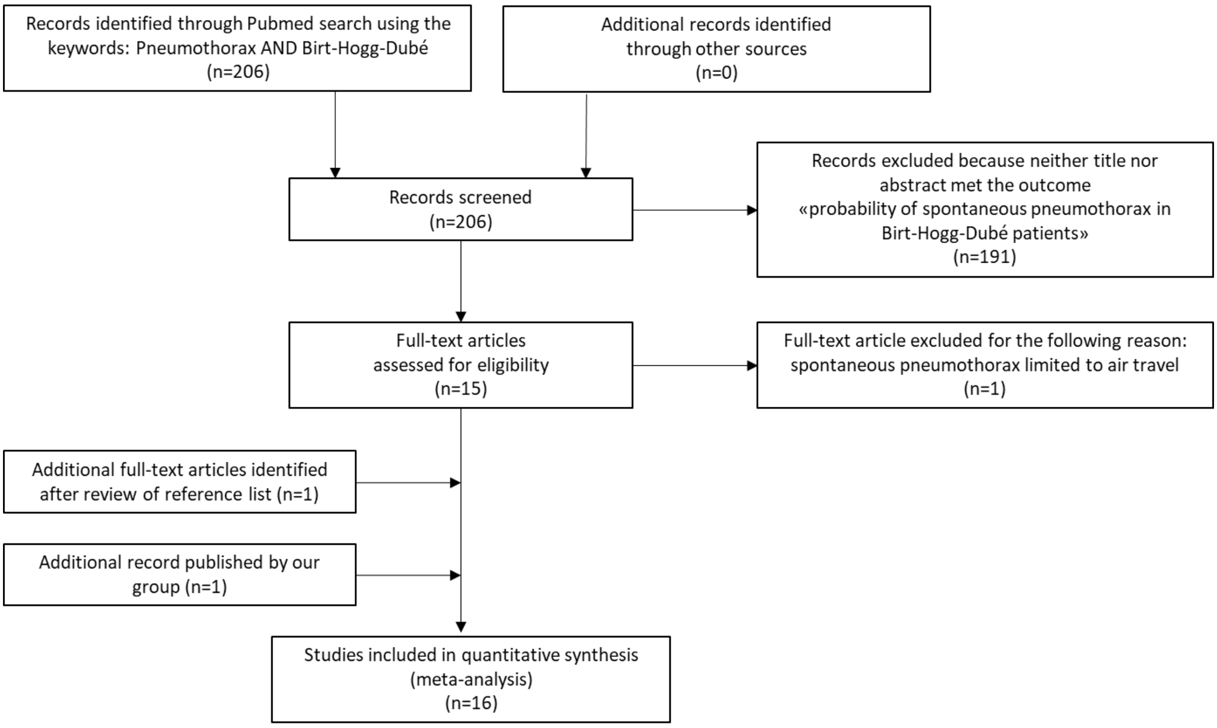

Flow diagram depicting the search strategy regarding probability of SP in BHD.

Supplementary Figure S7
A)

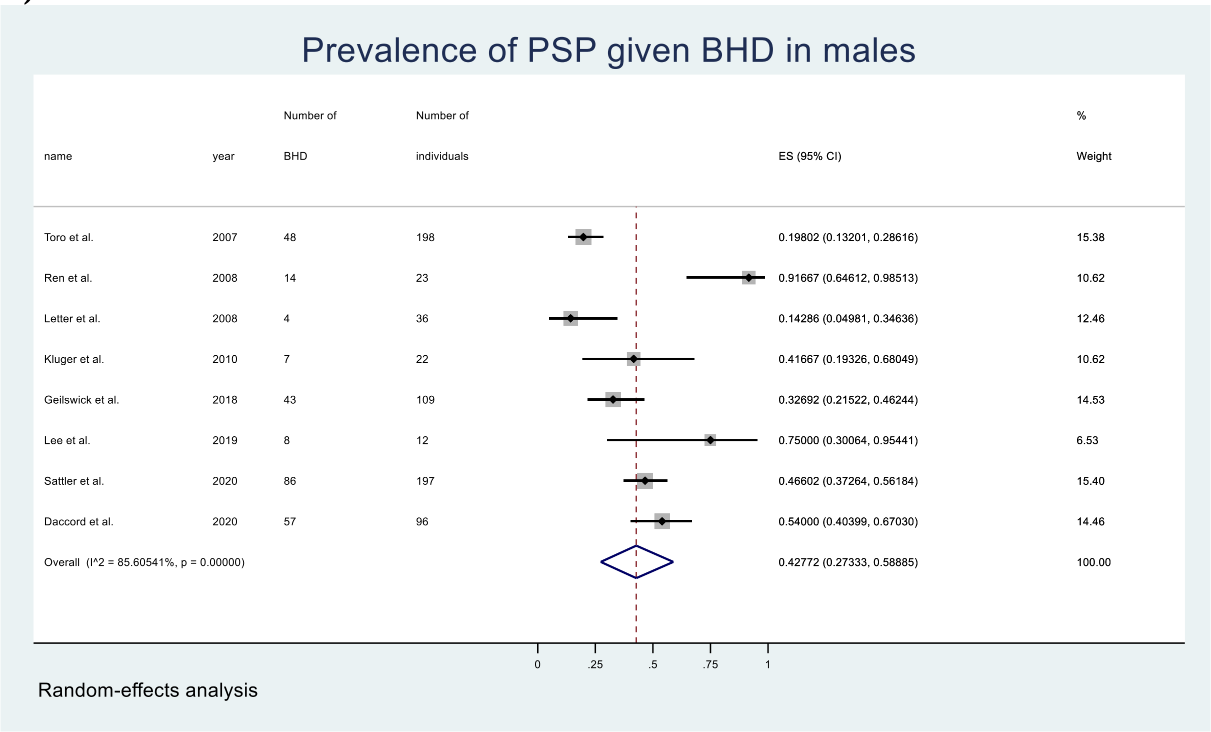

B)

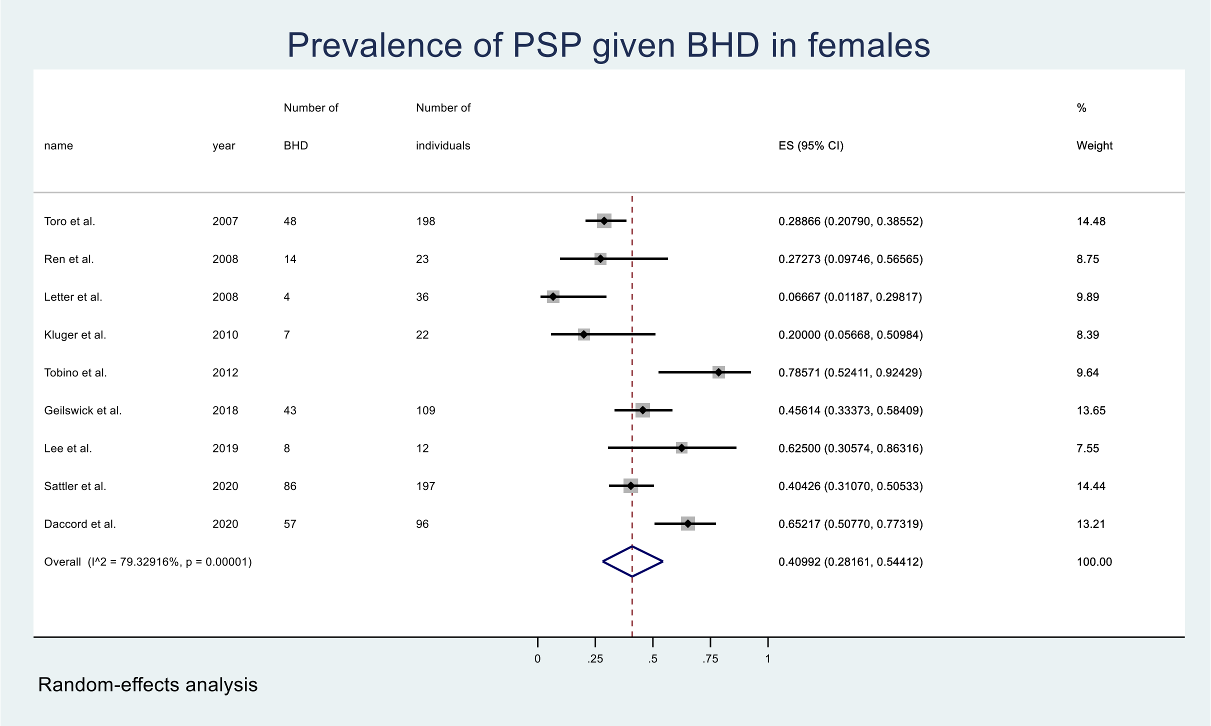

Forest plot of the prevalence of SP in BHD, random-effects model, stratified by gender (A, in males and B, in females). Due to software features, the values shown in this figure are slightly different from those of the text, as the between-study variance was calculated by the DerSimonian & Laird method, whereas in the text it was computed by restricted maximum likelihood.

Supplementary Table S1. Bayes equation's components estimated by fixed-effect models.

		women	men	all
Probability of BHD in apparent PSP (95% CI)		0.20 (0.14, 0.27)	0.05 (0.04, 0.07)	0.09 (0.07, 0.11)
Incidence rate of PSP in general population, per 100'000 person-years (95% CI)	overall	6.15 (6.08, 6.22)	21.02 (20.89, 21.15)	13.41 (13.34, 13.49)
	< 2000	3.10 (2.48, 3.88)	10.66 (9.51, 11.96)	6.72 (6.07, 7.44)
	> 2000	6.16 (6.09, 6.24)	21.06 (20.93, 21.19)	13.44 (13.37, 13.51)
Prevalence of PSP in the general population with PSP duration of 16 days, per 100'000 persons (95% CI)	overall	0.19 (0.18, 0.19)	0.79 (0.79, 0.80)	0.50 (0.49, 0.50)
	< 2000	0.10 (0.07, 0.12)	0.46 (0.41, 0.52)	0.28 (0.25, 0.31)
	> 2000	0.19 (0.19, 0.19)	0.80 (0.79, 0.80)	0.50 (0.49, 0.50)
Prevalence of PSP in the general population with PSP duration of 30 days, per 100'000 persons (95% CI)	overall	0.35 (0.35, 0.36)	1.49 (1.48, 1.50)	0.93 (0.92, 0.93)
	< 2000	0.18 (0.14, 0.23)	0.87 (0.77, 0.97)	0.53 (0.48, 0.59)
	> 2000	0.35 (0.35, 0.36)	1.49 (1.48, 1.50)	0.93 (0.93, 0.94)
Probability of occurrence of a SP in BHD (95% CI)		0.40 (0.35, 0.46)	0.37 (0.31, 0.42)	0.44 (0.42, 0.46)
Prevalence of BHD in the general population with PSP duration of 16 days, per million persons (95% CI)	overall	0.93 (0.64, 1.36)	1.16 (0.78, 1.72)	0.96 (0.75, 1.23)
	< 2000	0.48 (0.31, 0.76)	0.68 (0.45, 1.02)	0.55 (0.42, 0.72)
	> 2000	0.93 (0.64, 1.36)	1.16 (0.78, 1.72)	0.96 (0.75, 1.23)
Prevalence of BHD in the general population with PSP duration of 30 days, per million persons (95% CI)	overall	1.74 (1.20, 2.54)	2.17 (1.46, 3.22)	1.80 (1.41, 2.30)
	< 2000	0.90 (0.57, 1.42)	1.27 (0.84, 1.91)	1.03 (0.79, 1.35)
	> 2000	1.75 (1.20, 2.56)	2.18 (1.47, 3.23)	1.81 (1.41, 2.31)

95% CI: 95% confidence interval, PSP: primary spontaneous pneumothorax, SP: spontaneous pneumothorax, <2000: before year 2000, >2000: after year 2000.
